# Glycerol Kinase Gene Variant as a Cause of Pseudohypertriglyceridemia and Apparent Poor Response to Plozasiran

**DOI:** 10.1210/jcemcr/luaf146

**Published:** 2025-07-10

**Authors:** Miriam Larouche, Christie Ballantyne, Josiane Dufour, Diane Brisson, Bruce Given, Daniel Gaudet

**Affiliations:** Department of Medicine, Université de Montréal and ECOGENE-21, Chicoutimi, QC G7H 7K9, Canada; Internal Medicine, Baylor College of Medicine, Houston, TX 77046, USA; Department of Medicine, Université de Montréal and ECOGENE-21, Chicoutimi, QC G7H 7K9, Canada; Department of Medicine, Université de Montréal and ECOGENE-21, Chicoutimi, QC G7H 7K9, Canada; R & D Department, Arrowhead Pharmaceuticals, Pasadena, CA 91105, USA; Department of Medicine, Université de Montréal and ECOGENE-21, Chicoutimi, QC G7H 7K9, Canada

**Keywords:** glycerol kinase, hypertriglyceridemia, APOC3 inhibitor, siRNA, plozasiran

## Abstract

Severe hypertriglyceridemia (HTG) is characterized by plasma triglyceride (TG) levels >500 mg/dL (SI: 5.7 mmol/L) (reference range, <150 mg/dL [SI: <1.7 mmol/L]) and is linked to cardiovascular disease and pancreatitis risk. Treatment typically involves dietary restrictions and lipid-lowering medications. Glycerol kinase deficiency (GKD) is a rare genetic disorder that causes pseudo-HTG.

In SHASTA-2, a study of patients with severe HTG, most subjects (>90%) treated with plozasiran, an apolipoprotein C-III (APOC3) small interfering RNA (siRNA), achieved TG levels <500 mg/dL (SI: 5.7 mmol/L), below the risk threshold for acute pancreatitis. We report herein a case study of a 65-year-old male apparently not responding to plozasiran. The patient was shown to carry a loss-of-function variant in the *GK* gene resulting in GKD, with high free glycerol (40.24 mg/dL or 4.37 mmol/L) (reference range, 0.03-0.13 mmol/L) that contributed to an overestimation of TG concentration. After correcting for free glycerol, the patient was noted to have had mild HTG, with plozasiran treatment decreasing real TG values by up to 71%.

This case report suggests that in the absence of response to APOC3 inhibition, measuring free glycerol could be clinically relevant. It also highlights that APOC3 inhibition has no effect on free glycerol concentration.

## Introduction

Severe hypertriglyceridemia (HTG) is characterized by triglyceride (TG) levels >500 mg/dL (SI: 5.7 mmol/L) (reference range, <150 mg/dL [SI: <1.7 mmol/L]) [1]. Dissecting the underlying cause of severe HTG is mandatory to correctly evaluate the associated risks and establish the appropriate treatment plan. Most patients with severe HTG accumulate both chylomicrons and very-low density lipoproteins (VLDLs), conferring a higher risk for cardiovascular diseases [1]. When TG values are >880 mg/dL (SI: 10 mmol/L), clinically corresponding to chylomicronemia that could be intermittent or persistent, the risk of acute pancreatitis increases [2]. Chylomicrons, the largest circulating lipoproteins, are formed in the enterocytes following a meal to transport postprandial fat. Most patients with severe HTG combine genetic susceptibility and secondary causes including elements of the metabolic syndrome (obesity, type 2 diabetes) or other factors increasing TG levels [3].

Laboratory techniques that are clinically used to measure TG values are based on the measurement of glycerol, which binds to fatty acids to form acylglycerols and triglycerides. Most circulating glycerol molecules are thus bound to fatty acids so that free glycerol values are usually very low (less than 0.9 mg/dL or 0.1 mmol/L) (reference range, 0.03-0.13 mmol/L) and do not interfere with TG measurements [4]. It has been established that there was no need to routinely correct analytic enzymatic TG concentrations for free glycerol [4]. However, in rare circumstances, free glycerol values can be extremely elevated, hugely overestimating TG measures and leading to inaccurate diagnosis of severe HTG, and possibly biased evaluation of response to TG-lowering agents.

The key enzyme involved in glycerol metabolic pathways is glycerol kinase (GK). This enzyme is critical for the phosphorylation of glycerol in glycerol-3-phosphate and formation of TG [5]. Glycerol kinase deficiency (GKD) is an X-linked recessive disorder leading to hyperglycerolemia (glycerol values ranging from 18.4 mg/dL to 73.7 mg/dL (SI: 2.0 mmol/L to >8.0 mmol/L)) [6] and is often detected as pseudohypertriglyceridemia using common enzymatic methods of measurement. GKD is also associated with an increased risk of glucose intolerance [7, 8].

Several new or emerging therapies target severe HTG, including apolipoprotein C-III (APOC3) inhibitors, angiopoietin-like (ANGPTL) protein 3 or 3/8 inhibitors, ANGPTL4 inhibitors, fibroblast growth factor 21 (FGF21) analogs, and APOC2 mimetics. APOC3 inhibitors exhibit both lipoprotein lipase (LPL)-dependent and LPL-independent mechanisms of action and are effective in all spectrums of HTG [9, 10]. Plozasiran is an APOC3 small interfering RNA (siRNA) associated with a significant placebo-adjusted decrease in TG values in patients with HTG and mixed dyslipidemias in both phase 1 and 2 clinical trials [11-13]. The phase 2 SHASTA-2 study (NCT04720534) [12] involved patients with severe HTG. We present herein the case report of a participant who was apparently not responding to plozasiran, since measured TG levels remained elevated during the open-label extension period (up to 116 weeks post-randomization). This patient was investigated for defects in the glycerol metabolic pathway.

## Case Presentation

The patient was a 65-year-old man randomized in the SHASTA-2 study based on TG values >5.7 mmol/L (500 mg/dL) at screening. This patient never experienced any cardiovascular or acute pancreatitis event and his total cholesterol, low-density lipoprotein cholesterol (LDL-C), high-density lipoprotein cholesterol (HDL-C) concentrations, and lipoprotein(a) values were in the normal range. He was not overweight (body mass index 24.7 kg/m^2^), kept an active lifestyle, and did not have type 2 diabetes, glucose intolerance or fatty liver as assessed by ultrasonography and stiffness measurement (FibroScan^®^). He experienced recurrent ureteral calculi and renal colic in his late thirties. Despite the use of different TG-lowering therapies over the years, TG values in this patient remained elevated.

The patient was enrolled in the SHASTA-2 study in 2022 to receive the APOC3 siRNA plozasiran, based on a measured TG value of 601.8 mg/dL (SI: 6.8 mmol/L) at screening. The unblinding of the study in 2023 revealed that the patient received subcutaneous injections of placebo 10 mg on day 1 and week 12 and was followed through week 48. He then joined the 2-year open-label extension period in 2023 and received plozasiran 10 mg every 3 months for a total of 4 injections. The dose of plozasiran was then increased to 25 mg quarterly for the subsequent injections. Despite the administration of plozasiran, the patient's measured TG values remained elevated, ranging from 451.3 mg/dL to 531.0 mg/dL (SI: 5.1 to 6.0 mmol/L).

## Diagnostic Assessment

**Figure 1.**
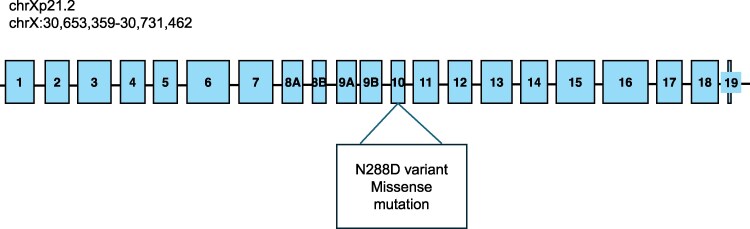
Depiction of the GK gene on chromosome Xp21.2 showing the loss-of-function variant (N288D missense mutation) known to cause glycerol kinase deficiency.

To investigate the apparent poor response to plozasiran, free glycerol was measured and *GK* genotyping performed, revealing that the patient carried a pathogenic loss-of-function variant in the *GK* gene (chrXp21.2) (Fig. 1) known to cause GKD (N288D variant) [7]. Accordingly, the free glycerol value was 40.24 mg/dL (SI: 4.37 mmol/L), which is 100-fold above normal, thus contributing to an important overestimation of real TG concentration. It is documented that free glycerol values vary little over time and have high heritability [7]. Since glycerol concentrations only fluctuated by 6% in this patient during the study, these results suggest that the inhibition of APOC3 has no or poor effect on free glycerol levels.

## Treatment

**Figure 2.**
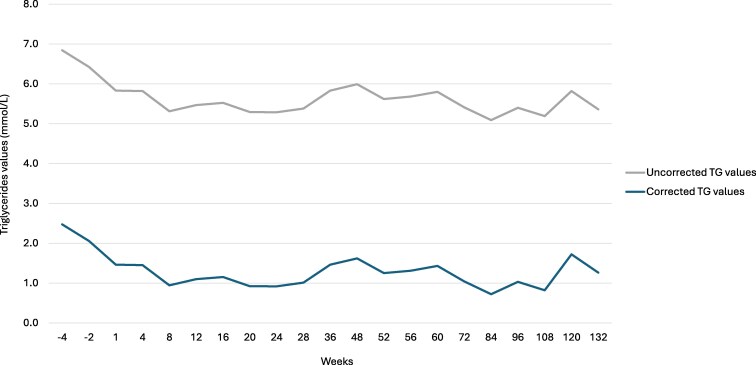
Plasma triglyceride (TG) values before and after correction for plasma free glycerol. The subject was a 65-year-old male individual with pseudohypertriglyceridemia caused by isolated glycerol kinase deficiency (GKD) treated with plozasiran.

After correcting for free glycerol, it was observed that the patient had only mild HTG and that plozasiran succeeded in decreasing real TG values by up to 71% (from 221.2 mg/dL to 62.0 mg/dL) (SI: 2.5 to 0.7 mmol/L) (Fig. 2). Following the diagnosis of GKD and treatment with plozasiran, no additional lipid-lowering agent was required.

## Outcome and Follow-Up

Correcting TG values for free glycerol concentrations revealed that the patient had mild HTG at screening. The administration of 25 mg plozasiran successfully decreased TG levels, but did not improve free glycerol concentration, suggesting that APOC3 inhibition has no effect on free glycerol.

## Discussion

Considering that APOC3 inhibitors exhibit both LPL-dependent and LPL-independent mechanisms of action, most patients with severe HTG, including those with persistent chylomicronemia, are likely to respond [14]. We report herein the case of a patient with falsely overestimated severe HTG who apparently did not respond to inhibition of APOC3 with plozasiran due to plasma accumulation of free glycerol. Techniques to assess plasma TG concentrations are based on the measurement of total plasma glycerol concentration, which includes free and acylglycerol-bound glycerol. Since plasma concentration of free glycerol only marginally contributes to TG concentrations (8.9 mg/dL or SI: <0.1 mmol/L) in the general population, correction is not routinely applied [4]. However, GKD is characterized by extremely elevated free glycerol concentrations, resulting in an overestimation of real TG levels and interfering with accurate assessment of the efficacy of TG-lowering interventions. Although poorly documented, the prevalence of GKD is estimated at 1 per million [15, 16]. In the presence of poor or mitigated response to TG-lowering therapies and despite the fact that GKD is a rare condition, it might be clinically worthwhile to measure free glycerol concentration. When free glycerol concentrations are significantly higher than the normal range, TG values should be corrected.

In the SHASTA-2 study, GKD was a cause of apparent poor response to plozasiran APOC3 inhibition [12]. The results highlighted in this case study are clinically important as the risks associated with severe HTG and GKD differ. Patients affected by GKD are not at high risk of HTG-induced acute pancreatitis. However, glycerol is at the interface of lipid and glucose metabolism, and it has been reported that free glycerol concentration correlates with 2-hour glycemia after an oral glucose tolerance test in GKD as well as in the general population [7]. The risk of glucose intolerance or type 2 diabetes should thus be monitored more frequently in GKD patients.

Before being diagnosed adequately, affected patients with GKD and severe HTG have most likely been treated with several lipid-lowering agents and restrictive diets over the years, with no response to such interventions and importantly, with increasing anxiety and negative impact on overall quality of life. Several case reports have exposed these issues [16-19]. In the presence of GKD, TG-lowering interventions are not always necessary since patients are facing pseudo-HTG that overestimates the real TG values [16-19].

This case report suggests that in the absence of response to APOC3 inhibition, measuring free glycerol could be clinically relevant. It also highlights that APOC3 inhibition has no effect on free glycerol concentration. This has been revealed by exposing a patient presenting a rare disease associated with extreme hyperglycerolemia to plozasiran. This case report also provides additional evidence to support the hypotheses that potent inhibition of APOC3 with plozasiran is expected to have a complete response in reducing plasma TGs through both LPL-dependent and LPL-independent pathways.

## Learning Points

- Treatment for severe HTG, which carries a high risk for atherosclerotic cardiovascular disease, metabolic syndrome, and acute pancreatitis, generally includes long-term dietary restrictions and aggressive lipid-lowering treatment.
- This report highlights the case of a patient with falsely elevated TG levels (pseudo-HTG) caused by glycerol kinase deficiency (GKD) who appeared not to benefit from TG-lowering treatment with plozasiran, an APOC3 siRNA.
- When TG-lowering therapies are unsuccessful, measurement of free glycerol concentration to rule out GKD and pseudo-HTG could be clinically relevant.
- The case also suggests that APOC3 inhibition has no effect on free glycerol levels.

## Data Availability

Some or all datasets generated during and/or analyzed during the current study are not publicly available but are available from the corresponding author on reasonable request.
